# An early combination of concurrent chemoradiotherapy with immune checkpoint inhibitors for cervical cancer is superior to a late combination: a propensity-score matching analysis

**DOI:** 10.3389/fonc.2024.1429176

**Published:** 2024-11-21

**Authors:** Yi-Ming Ma, Shu-Xia Cheng, Ming-Chuan Zhang, Hui-Ying Zhang, Jun-Jiao Gu, Pan-Pan Zhao, Hong Ge

**Affiliations:** ^1^ Department of Gynecologic Oncology, The Affiliated Cancer Hospital of Zhengzhou University and Henan Cancer Hospital, Zhengzhou, China; ^2^ Department of Oncology, First Affiliated Hospital of Henan University, Kaifeng, China; ^3^ Department of Oncology, Huaihe Hospital of Henan University, Kaifeng, China; ^4^ Department of Gynecologic, First Affiliated Hospital of Henan University of Science and Technology, Jiaozuo, China; ^5^ Department of Radiation Oncology, The Affiliated Cancer Hospital of Zhengzhou University and Henan Cancer Hospital, Zhengzhou, China

**Keywords:** immune checkpoint inhibitors, cervical cancer, application timing, progression-free survival, objective response rate, treatment-related adverse events

## Abstract

**Purpose:**

This study compared the timing effects of immune checkpoint inhibitor (ICIs) administration on the efficacy and safety of concurrent chemoradiotherapy for cervical cancer.

**Methods:**

This study included patients with advanced cervical cancer who received concurrent chemoradiotherapy with ICIs. The patients were divided into early-application (n=51) and late-application groups (n=56) according to the ICI application timing. The primary objective was assessing progression-free survival (PFS) and its associated factors; secondary objectives included assessing objective remission rates (ORR) and treatment-related adverse events (TRAEs).

**Results:**

Before propensity score matching (PSM), the median PFS (mPFS) times were significantly different: 11.5 months (95% CI: 11.0–13.2) and 7.5 months (95% CI: 6.5–9.0) for the early and late groups, respectively (P<0.001). After PSM, the mPFS times remained significantly different: 11.5 months (95% CI: 11.0–13.8) and 6.5 months (95% CI: 6.1–9.0), respectively (P<0.001). The PSM tumor-response ORR in the early combination group (74.3%) was significantly greater than the 31.4% in the late combination group (P<0.001). After PSM, multivariate Cox analysis showed tumor diameter (P=0.004), distant organ metastasis (P=0.047), and timing of combination therapy (P<0.001) were independently associated factors affecting PFS. The most common TRAEs in the two groups of patients were neutropenia, nausea and vomiting, and fatigue, with no significant difference in incidence (P>0.050).All adverse reactions were resolved, and no adverse reaction-related deaths occurred.

**Conclusion:**

In patients with cervical cancer treated with concurrent chemoradiotherapy, earlier immunotherapy improves survival and is equivalent in safety to ICIs late application.

## 1 Introduction

Cervical cancer is closely related to persistent infection with the human papillomavirus (HPV), and the occurrence and development of tumors are related to immune suppression. Therefore, the role of immune factors in cervical cancer cannot be ignored. Immune checkpoints are a current research focus in the treatment of malignant tumors. Programmed cell death receptor 1 (PD-1) binds to programmed cell death receptor ligand 1 (PD-L1), which promotes the immune escape of tumors by inhibiting the activity and function of natural killer cells and T lymphocytes, thus promoting the development and progression of various malignancies. Immune checkpoint inhibitors (ICIs) can block the interaction between PD-1 and PD-L1, promote the immune system to kill tumor cells (1, 2), and have been proven to be effective in treating metastatic and/or recurrent cervical cancer. However, few reports have been written on the timing of the application of ICIs in concurrent chemoradiotherapy for cervical cancer. Therefore, this study compared the effect of different combinations of concurrent chemoradiotherapy and ICIs on the efficacy and safety of cervical cancer through a cohort study and analyzed the prognostic factors.

## 2 Materials and methods

### 2.1 Patient information

This study included 107 patients with cervical cancer who were treated from March 2020 to September 2022 at the Zhengzhou University Affiliated Cancer Hospital, Henan University Huaihe Hospital, Henan University First Affiliated Hospital, and Henan University of Technology First Affiliated Hospital. All patients signed an informed consent form and received concurrent chemoradiotherapy and immune checkpoint inhibitors (ICIs).

### 2.2 Inclusion and exclusion criteria

**Figure 1:**
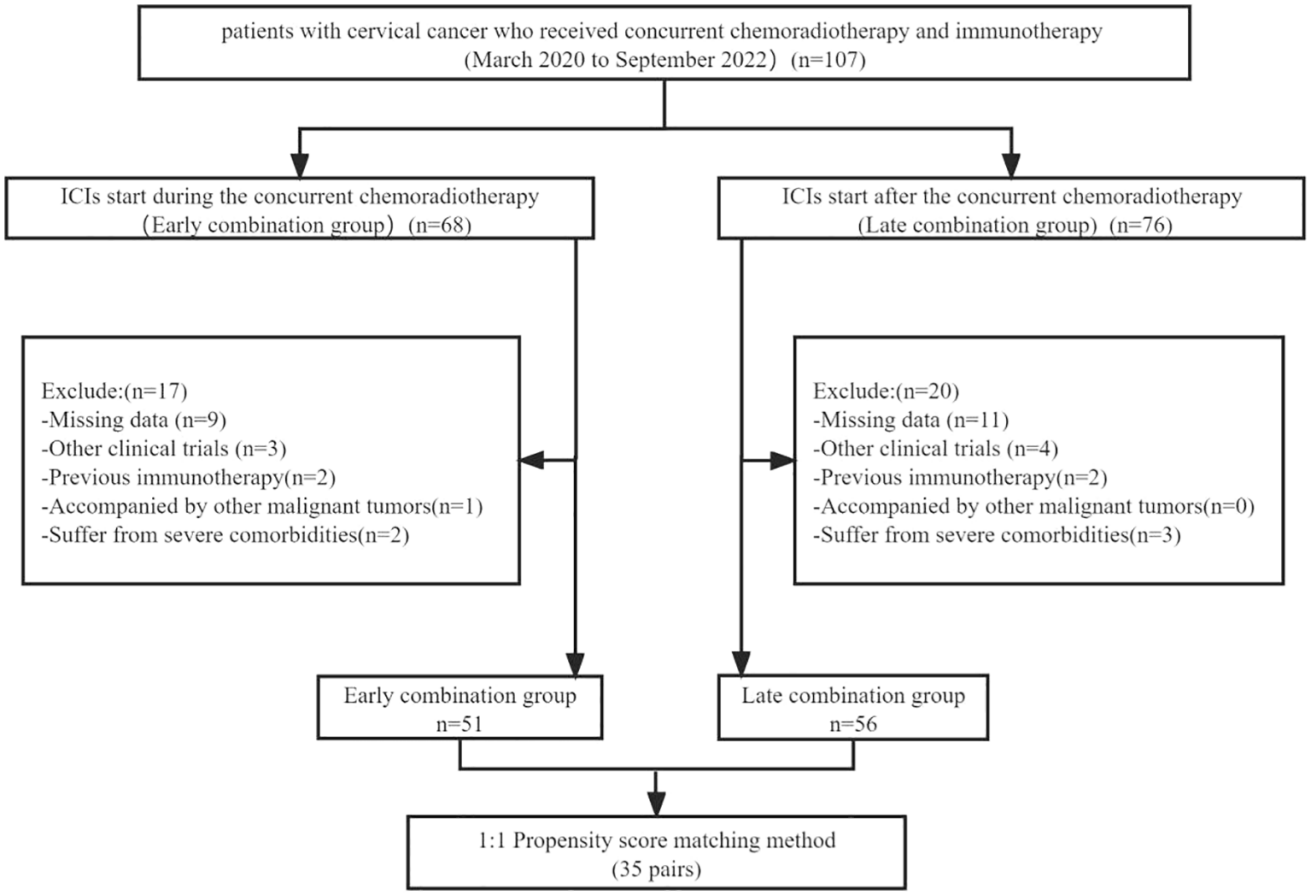
Flow diagram for patient screening.

The inclusion criteria were (1) age between 18 and 75 years, (2) cervical cancer was confirmed via histopathological examination, (3) Eastern Collaborative Oncology Group (ECOG) physical status score of 0 or 1, and (4) at least one measurable target lesion should be evaluated according to the Response Evaluation Criteria in Solid Tumors (RECIST version 1.1). The exclusion criteria were (1) patients with other malignant tumors, (2) patients who have received Immunotherapies, (3) clinical trial participation, (4) patients with incomplete data, and (5) severe comorbidities, including cardiac, pulmonary, renal, and coagulation dysfunction (**Figure 1**).

### 2.3 Treatment regimens

Patients in both groups underwent a radical concurrent chemoradiotherapy regimen: linear accelerator 6 MV X-ray abdominopelvic intensity-modulated radiation therapy, 45 Gy/para-aortic lymph nodes, 50 Gy/pelvic lymph nodes 55 Gy/25 f; or pelvic intensity-modulated radiation therapy, 45 Gy/pelvic lymph nodes, 55 Gy/25 f. Afterward, 10–15 Gy of supplementary radiation therapy was administered to enlarged lymph nodes and para-uterine tissues (192Ir 3D intracavitary brachytherapy 30 Gy/5f). Radiotherapy was initiated with concurrent cisplatin (Qilu Pharmaceutical; Jinan, China) sensitization chemotherapy (40 mg/m**^2^** ivgtt) once a week for 5 weeks.

In this study, the ICIs (200 mg, ivgtt, once every 3 weeks, with six infusions) included sintilimab (Innovent Pharmaceutical; Suzhou, China), tislelizumab (BeiGene Pharmaceutical, Shanghai, China), and camrelizumab (Hengrui Pharmaceutical, Lianyungang, China). The early-combination group starts during the concurrent chemoradiotherapy.; the late-stage combination group starts after the concurrent chemoradiotherapy. The timing of the application of ICIs is determined by both the physician and the patient in the real world. In the event that a grade 3 or higher adverse event was judged to be associated with ICIs, the drug would be suspended until the adverse event is mitigated or eliminated.

### 2.4 Study endpoints

The primary objective of this study was to assess progression-free survival (PFS) and its associated factors, and the secondary objectives were to assess objective remission rates (ORR) and treatment-related adverse events (TRAEs).

### 2.5 Assessment and follow-up

Follow-up visits are required every 4–6 weeks after the end of treatment and include imaging with pelvic plain and dynamic enhancement magnetic resonance imaging and enhanced computed tomography of the neck, chest, and abdomen. Two experienced radiologists measured the size of lesions at the same site and evaluated the tumor response based on RECIST version 1.1, including complete response (CR), partial response (PR), stable disease (SD), and progressive disease(PD). The ORR was defined as the proportion of patients with CR+PR. PFS was defined as the time interval from the date of treatment initiation to the date of detection of disease progression or death from any cause. Overall survival (OS) was defined as the time interval from the date of treatment initiation to the date of death from any cause, and adverse events were evaluated through patient laboratory examinations, telephone follow-up, and medical history records using the National Cancer Institute (NCI) Common Terminology for Adverse Events (CTCAE) version 5.0. All patients were followed-up until March 2023.

### 2.6 Statistical analysis

To reduce patient selection bias and balance the variables between the two groups, we performed PSM analysis using a 1:1 ratio to construct a balanced cohort with a calliper value of 0.1. Baseline variables included the patient’s age, ECOG score, hemoglobin, PD-L1 expression status, squamous cell carcinoma associated antigen (SCC-Ag), distant metastasis, lymph node metastasis, maximum tumor diameter, HPV infection, pathology type, and previous treatment.

Categorical variables are presented as percentages and were calculated using the chi-squared test; continuous variables are expressed as the mean ± standard deviation and were calculated using Student’s t-test. The Kaplan–Meier method was used to evaluate the differences in PFS and OS between the two groups. Univariate analysis was used to assess the statistical significance of clinical characteristics. Statistically, significant variables were included in the analysis using a multifactorial Cox regression model to identify predictors associated with PFS. A P-value < 0.05 was considered statistically significant. All analyses were performed using R software (version 4.1.2; R Foundation for Statistical Computing, Vienna, Austria; https://www.r-project.org/).

## 3 Results

### 3.1 Patient characteristics

In total, 107 patients with advanced cervical cancer who received immunotherapy were included in this study. According to the inclusion and exclusion criteria, 51 cases were classified into the early combination group and 56 cases into the late combination group. The baseline data of the two groups of patients showed varying degrees of difference, with a significant difference in lymph node metastasis between the two groups (P<0.05), which did not achieve sufficient balance. After PSM analysis, the differences in covariates between the two groups were not statistically significant (P>0.05). A comparison of the clinicopathological parameters between the two groups of patients before and after PSM is shown in **Table 1**.

**Table 1:** Patient characteristics before PSM adjustment and after PSM adjustment.

Variable	Before PSM adjustment				After PSM adjustment			
	Early combination group (n=51)	Late combination group (n=56)	*P* value	SMD	Early combination group (n=35)	Late combination group (n=35)	*P* value	SMD
Age (years)								
<50	21 (41.2)	22 (39.3)	0.999	0.039	11 (31.4)	14 (40.0)	0.618	0.180
≥50	30 (58.8)	34 (60.7)			24 (68.6)	21 (60.0)		
Hb (g/L)								
<100	40 (78.4)	43 (76.8)	1.000	0.039	29 (82.9)	27 (77.1)	0.765	0.143
≥100	11 (21.6)	13 (23.2)			6 (17.1)	8 (22.9)		
Organ metastasis								
None	14 (27.5)	26 (46.4)	0.068	0.401	13 (37.1)	16 (45.7)	0.627	0.175
Have	37 (72.5)	30 (53.6)			22 (62.9)	19 (54.3)		
PD-L1								
PD-L1 <1	30 (58.8)	32 (57.1)	0.965	0.052	20 (57.1)	21 (60.0)	0.642	0.227
1≤PD-L1<10	9 (17.6)	11 (19.6)			5 (14.3)	7 (20.0)		
PD-L1≥10	12 (23.5)	13 (23.2)			10 (28.6)	7 (20.0)		
SCC-Ag								
<2.7	23 (45.1)	24 (42.9)	0.969	0.045	14 (40.0)	15 (42.9)	1.000	0.058
≥2.7	28 (54.9)	32 (57.1)			21 (60.0)	20 (57.1)		
Size								
<4 cm	19 (37.3)	32 (57.1)	0.062	0.407	18 (51.4)	18 (51.4)	1.000	<0.001
≥4 cm	32 (62.7)	24 (42.9)			17 (48.6)	17 (48.6)		
HPV								
Infection	22 (43.1)	25 (44.6)	1.000	0.030	15 (42.9)	13 (37.1)	0.807	0.117
No infection	29 (56.9)	31 (55.4)			20 (57.1)	22 (62.9)		
ECOG								
0	28 (54.9)	32 (57.1)	0.969	0.045	17 (48.6)	18 (51.4)	1.000	0.057
1	23 (45.1)	24 (42.9)			18 (51.4)	17 (48.6)		
Lymph metastasis								
None	17 (33.3)	8 (14.3)	0.036	0.459	7 (20.0)	6 (17.1)	1.000	0.074
Have	34 (66.7)	48 (85.7)			28 (80.0)	29 (82.9)		
Histology								
Squamous cell carcinoma	41 (80.4)	45 (80.4)	1.000	0.001	28 (80.0)	30 (85.7)	0.751	0.152
Adenocarcinoma	10 (19.6)	11 (19.6)			7 (20.0)	5 (14.3)		
Previously treated								
None	32 (62.7)	34 (60.7)	0.987	0.042	21 (60.0)	23 (65.7)	0.805	0.118
Have	19 (37.3)	22 (39.3)			14 (40.0)	12 (34.3)		

PSM, propensity score matching; SMD, standardized mean difference; SCC-Ag, sguamous cell carcinoma associated antigen; Hb, hemoglobin; HPV, human papillomavirus; ECOG, Eastern Collaborative Oncology Group.

### 3.2 Survival analysis

**Figure 2:**
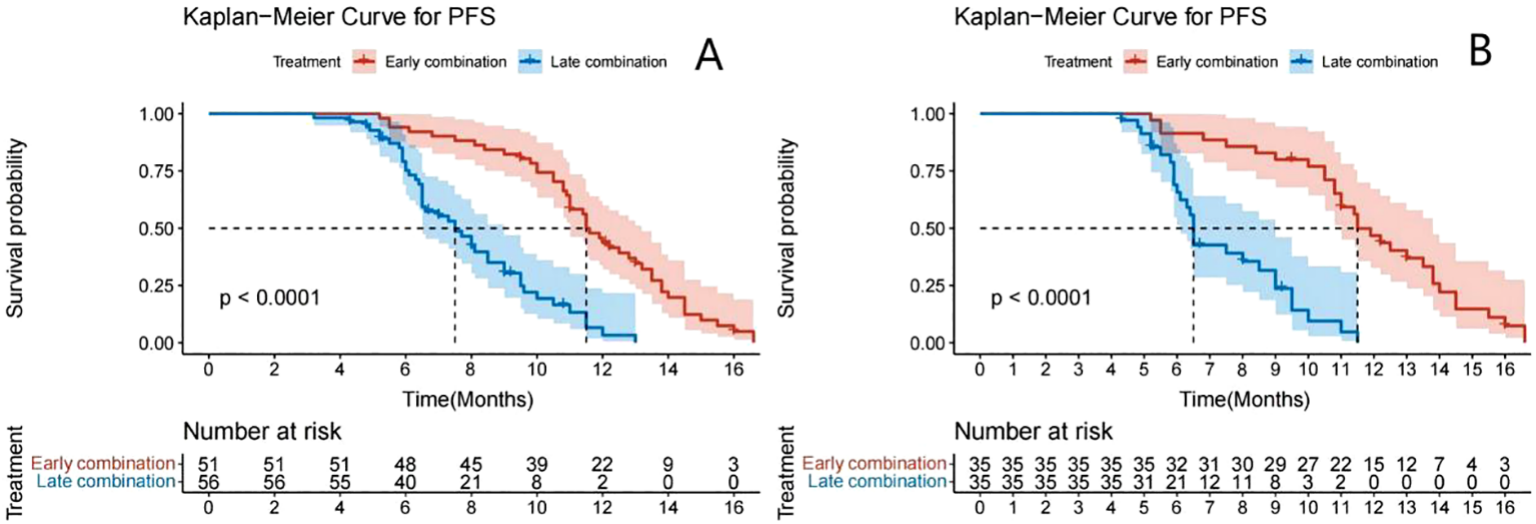
Kaplan–Meier analyses of PFS according to early combination group and late combination group before **(A)** and after **(B)** PSM.

**Figure 3:**
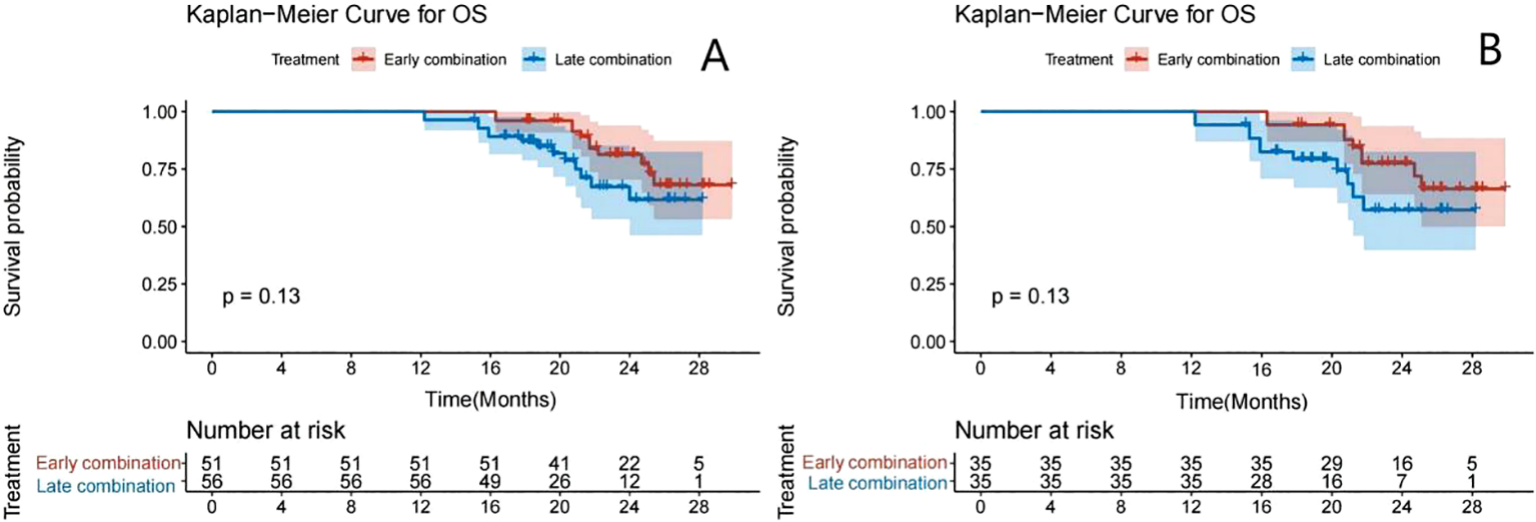
Kaplan–Meier analyses of OS according to the early- and late-combination groups before **(A)** and after **(B)** PSM.

The Kaplan–Meier survival curve showed that the median PFS (mPFS) before PSM was 11.5 months (95% CI: 11.0–13.2) and 7.5 months (95% CI: 6.5–9.0) for the early and late stages, respectively. A significant difference was found in the mPFS between the two groups (P<0.001) (**Figure 2A**). After PSM, the mPFS of the two groups were 11.5 months (95% CI: 11.0–13.8) and 6.5 months (95% CI: 6.1–9.0), respectively. A significant difference was also found in mPFS between the two groups (P<0.001) (**Figure 2B**). The median OS was not achieved (**Figure 3**).

### 3.3 Tumor response

Before PSM analysis, the ORR of the early and late combination groups were significantly different: 76.5% and 32.1%, respectively (P<0.001). After PSM analysis, the ORR of tumor response in the early combination group was 74.3%, significantly greater than that in the late combination group (31.4%; P=0.001) (**Table 2**).

**Table 2:** Tumor response before and after PSM analysis assessed using RECIST criteria.

Tumor response	Before PSM		P value	After PSM		P value
	Early combination group	Late combination group		Early combination group	Late combination group	
CR	21 (41.2)	7 (12.5)		16 (45.7)	4 (11.4)	
PR	18 (35.3)	11 (19.6)		10 (28.6)	7 (20.0)	
SD	10 (19.6)	24 (42.9)		7 (20.0)	16 (45.7)	
PD	2 (3.9)	14 (25.0)		2 (5.7)	8 (22.9)	
ORR (CR+PR)	76.50%	32.10%	<0.001	74.30%	31.40%	0.001

PSM, propensity score matching; CR, Complete response; PR, Partial response; SD, Stable disease; PD, Progressive disease; ORR, Overall response rate.

### 3.4 Analysis of factors affecting PFS

Univariate and multifactorial Cox analyses were used to determine the prognostic factors affecting PFS. Before PSM analysis, PD-L1 expression, tumor diameter, organ metastasis, and timing of combination therapy were prognostic factors affecting PFS (P<0.050). Multivariate Cox analysis showed that tumor diameter (P=0.015) and timing of combination therapy (p<0.001) were related independently to PFS.

After PSM analysis, univariate analysis showed that tumor diameter, organ metastasis, and timing of combination therapy were prognostic factors affecting PFS (P<0.050). Multivariate Cox analysis showed that tumor diameter (P=0.004), distant organ metastasis (P=0.047), and timing of combination therapy (P<0.001) were independent factors affecting PFS (**Table 3**).

**Table 3:** Univariate and multivariate analysis of PFS.

Risk factor	Before PSM cohort				After PSM cohort			
	Univariate Analysis		Multivariate Analysis		Univariate Analysis		Multivariate Analysis	
	HR (95% CI)	p-value	HR (95% CI)	p-value	HR (95% CI)	P-value	HR (95% CI)	P-value
Age	1.006 (0.66–01.535)	0.976			0.730 (0.429–1.242)	0.246		
Hb	1.162 (0.705–1.917)	0.555			1.228 (0.646–2.333)	0.531		
PD-L1	0.882 (0.493–1.579)	0.673	0.687 (0.373–1.264)	0.228	0.650 (0.299–1.413)	0.277		
	1.712 (1.019–2.877)	0.042	0.722 (0.396–1.316)	0.287	1.855 (0.988–3.480)	0.054		
SCC-Ag	0.978 (0.641–1.493)	0.918			0.841 (0.495–1.430)	0.523		
HPV	1.275 (0.826–1.967)	0.272			1.501 (0.859–2.622)	0.154		
ECOG	0.880 (0.575–1.345)	0.554			0.725 (0.430–1.223)	0.228		
Size	1.866 (1.199–2.904)	0.006	1.904 (1.134–3.195)	0.015	3.135 (1.691–5.812)	<0.001	2.538 (0.355–4.753)	0.004
Lymph metastasis	1.344 (0.827–2.184)	0.232			0.974 (0.502–1.888)	0.938		
Organ metastasis	1.599 (1.022–2.503)	0.040	1.464 (0.878–2.443)	0.144	1.916 (1.105–3.322)	0.021	1.784 (1.009–3.156)	0.047
Histology	0.657 (0.374–1.153)	0.143			0.494 (0.230–1.064)	0.072		
timing of therapy	4.419 (2.683–7.278)	<0.001	5.911 (3.352–10.422)	<0.001	6.307 (3.198–12.441)	<0.001	5.716 (3.020–10.992)	<0.001
Previously treated	1.201 (0.780–1.848)	0.406			1.198 (0.695–2.066)	0.516		

PSM, propensity score matching; HR, hazard ratio; CI, confidence interval; SCC-Ag, squamous cell carcinoma associated antigen; HPV, human papillomavirus; ECOG, Eastern Collaborative Oncology Group.

### 3.5 Treatment-related adverse events

During treatment and follow-up, the most common TRAEs in the two groups of patients were neutropenia, nausea and vomiting, and fatigue, all of which improved after symptomatic treatment; the remaining most common events were decreased appetite and radiation proctitis. The most common grade 3 or above adverse events in the early combination group were neutropenia (11.8%) and fatigue (3.9%). In comparison, the most common grade 3 or above adverse event in the late combination group was nausea and vomiting (3.6%). No significant difference was found in the incidence of TRAEs at or above grade 3 between the two groups of patients (P>0.050). After symptomatic management and dose reduction or discontinuation, all TRAEs resolved. In the advanced combination group, 1 patient (1.8%) developed reactive capillary hyperplasia (RCCEP), 5 patients (8.9%) developed hypothyroidism, and 2 patients (3.6%) developed immune-related hepatitis, 1 case (1.8%) developed immune-related pneumonia, all of which were graded 1–2. After symptomatic treatment, they were relieved, and no deaths were related to adverse reactions (**Table 4**).

**Table 4:** Treatment-related adverse events between the two groups.

Adverse events	Early combination group (n=51)		Late combination group (n=56)	
	Any grade	≥ Grade 3	Any grade	≥ Grade 3
Neutropenia	32(62.7%)	6(11.8%)	17(30.4%)	0
Fatigue	28(54.9%)	2(3.9%)	19(33.9%)	1(1.8%)
Decreased appetite	21(41.2%)	0	13(23.2%)	0
Nausea and Vomiting	31(60.8%)	1(2.0%)	16(28.6%)	2(3.6%)
Radiation proctitis	20(39.2%)	1(2.0%)	14(25%)	1(1.8%)
Fever	2(3.9%)	0	1(1.8%)	0
Pain	5(9.8%)	0	3(5.4%)	0
Thrombocytopenia	5(9.8%)	0	1(1.8%)	0
Immune-related hepatitis	4(7.8%)	0	2(3.6%)	0
Rash	3(5.9%)	0	0(0.0%)	0
Gastrointestinal reaction	10(19.6%)	0	7(12.5%)	0
RCCEP	0	0	1(1.8%)	0
Hypothyroidism	0	0	5(8.9%)	0
Immune associated pneumonia	0	0	1(1.8%)	0

RCCEP, Reactive cutaneous capillary endothelial proliferation.

## 4 Discussion

In recent years, immune checkpoint inhibitors targeting PD-1/PD-L1 have played an important role as anti-tumor agents in advanced cervical cancer (3). However, most studies have focused on the efficacy of ICIs in recurrent metastatic cervical cancer, and relatively few reports have been written on the timing of ICIs application. This study found that immunotherapy advancement improves patient survival and is equivalent in safety to the late application of ICIs for cervical cancer patients treated with concurrent chemoradiotherapy and that tumor diameter, distant organ metastasis, and timing of combination therapy are independent risk factors affecting the efficacy of ICIs.

The 5-year survival rates of patients with advanced and recurrent cervical cancer are less than 20% and < 5%, respectively (4). Therefore, effective methods for treating advanced cervical cancer are lacking, and finding effective new treatment strategies is necessary. With the continuous development of tumor biology, the immune escape mechanism of tumors has attracted much attention during the process of tumorigenesis and development. Some studies have reported that inhibiting tumor-cell immune escape by enhancing the immune response can improve patient prognoses (5). Because of the unusual gene expression characteristics and molecular markers of tumor cells, the immune system can recognize and kill tumor cells during the body’s self-protection process. However, in the tumor microenvironment, T cells usually produce immune escape or immune tolerance because of over-inhibition, thus allowing tumor cells to escape from the surveillance of the immune system (6). Therefore, enhancing the immune response by suppressing the negative regulation of the immune system can inhibit the occurrence and development of tumors.

The National Comprehensive Cancer Network (NCCN) guidelines for cervical cancer (Version 1.2022) recommend pembrolizumab combined with chemotherapy as a first-line recommendation for patients with PD-L1 positive, recurrent, or metastatic cervical cancer. Sevko et al. (7)(2013) found that cyclophosphamide, platinum, or paclitaxel analogues could cause immunosuppression by reducing regulatory T cells and increasing the expression of the CD8^+^ T cell surface receptor PD-1 in the tumor microenvironment. Shaverdian et al. (8) found that radiotherapy combined with ICIs significantly prolongs the OS and PFS of patients with non-small cell lung cancer. Mayadev et al. (9)reported the research results of NRG GY-17 (NCT03738228) at the Society of Gynecological Oncology (SGO) Annual Meeting 2022. This study explored the efficacy of concurrent chemoradiotherapy combined with atezolizumab administered at different times in patients with locally advanced and high-risk cervical cancer. It showed that atezolizumab administered as pre-excitation therapy before chemoradiotherapy (on days −21, 0, and 21) achieved better tumor response rates (CR%: 45% *vs*. 27%; ORR%: 82% *vs*. 36%) than synchronous administration (on days 0, 21, and 42). Our research analysis showed that the median PFS was 11.5 months for the early application of ICIs group and 7.5 months for the late application of ICIs therapy after PSM and that early application of ICIs in radiotherapy or chemotherapy for advanced cervical cancer can improve patient survival, possibly because of the following reasons: (1) cytotoxic chemotherapeutic agents can activate the immune system, and its mechanism may be related to enhancing the immunogenicity of tumor cells (10); (2) radiotherapy causes local tumor cell death and induces changes in the tumor microenvironment, causing the release of large amounts of tumor immunogenic antigens, which can lead to immunogenic death and local immune responses (11–13).

Univariate analysis showed that tumor diameter, organ metastasis, and timing of combination therapy were prognostic factors affecting PFS (P<0.050). Multivariate Cox analysis showed that tumor diameter, distant organ metastasis, and timing of combination therapy were independent factors affecting PFS (P<0.050). The EMPOWER-Cervical 1/GOG-3016/ENGOT-cx9 study at the 2021 Annual European Society for Medical Oncology (ESMO) Congress noted that the effect of histologic staging (squamous cell carcinoma and adenocarcinoma, including adenosquamous carcinoma) made no significant difference on survival, consistent with our result that histologic type was not a prognostic factor for PFS. In addition, PD-L1 was not a relevant factor for prognosis in our study, consistent with the research results of Gibney et al. (14); that is, the expression of PD-L1 was not associated with OS or PFS in patients with advanced cervical cancer. Although studies have shown the expression of PD-L1 is associated with the prognoses of patients with various malignant tumors (15), the relationship between the expression level of PD-L1 and prognosis remains controversial. For example, Sznurkowski et al. (16) noted that PD-L1 expression in immune cells improved the prognosis of vulvar squamous carcinoma; however, Karpathiou et al. (17) concluded it did not affect the prognosis of laryngeal squamous cell carcinoma. In addition, Kim et al. (18) showed that PD-L1 is an effective target in the treatment of cervical squamous cell carcinoma. However, for patients with cervical adenocarcinoma, PD-L1 positive expression is associated with a lower survival rate. One possible reason for this result is that the evaluation criteria for PD-L1 expression have not yet been unified (19). Some studies (20) used PD-L1 >50% as the critical expression point; others (21) used PD-L1 >5% as the positive expression criterion; therefore, further exploration is needed.

The results of our study showed that, during the treatment and follow-up periods, the most common TRAEs in the two groups of patients were neutropenia, nausea and vomiting, and fatigue, all of which improved after symptomatic treatment; the remaining most common events were decreased appetite and radiation proctitis. No significant difference was found in the incidence of TRAEs between the two groups (P>0.050). Our study’s incidence of adverse events was consistent with previous studies (22, 23). The most common adverse events of any grade associated with pembrolizumab in the study by Colombo et al. (24) were anemia (61.2%), alopecia (56.4%), and nausea (39.7%); the most common grade 3–5 adverse events were anemia (30.3%) and neutropenia (12.4%). In our study, the most common grade 3 or above adverse events in the early combination group were neutropenia (11.8%) and fatigue (3.9%). In comparison, the most common grade 3 or above adverse event in the late combination group was nausea and vomiting (3.6%).The two groups’ most common grade 3 or greater adverse events differed primarily because of the potential additional toxicity during early combination chemotherapy or targeted therapy. All patients who experienced adverse events had their associated symptoms resolved or eliminated after symptomatic treatment or temporary interruption of dosing. Therefore, the results of our study suggest that the early application of ICIs for advanced cervical cancer is equivalent in safety to the late application of ICIs.

Some limitations need to be considered for our study. First, although PSM was performed, it cannot eliminate the possibility of data bias. Second, a longer follow-up period would benefit this study; the next step will be to validate our findings using OS as the endpoint. Finally, because of the need for prevention and control of the coronavirus epidemic, some patients may not have been able to receive treatment regularly, ultimately affecting the prognosis. Therefore, the findings of this study need to be further confirmed by a multicenter, large-sample, prospective, randomized controlled clinical study.

## 5 Conclusion

In summary, immunotherapy advancement improves survival for cervical cancer patients treated with concurrent chemoradiotherapy and is equivalent in safety to the late application of ICIs. However, further prospective studies are needed to explore and validate the optimal timing of ICIs application to provide a theoretical basis for achieving survival benefits for patients with advanced cervical cancer.

## Data Availability

The raw data supporting the conclusions of this article will be made available by the authors, without undue reservation.
